# Pediatric myelin oligodendrocyte glycoprotein antibody-associated disease in southern China: analysis of 93 cases

**DOI:** 10.3389/fimmu.2023.1162647

**Published:** 2023-06-02

**Authors:** Xiaojing Li, Wenlin Wu, Chi Hou, Yiru Zeng, Wenxiao Wu, Lianfeng Chen, Yinting Liao, Haixia Zhu, Yang Tian, Bingwei Peng, Kelu Zheng, Kaili Shi, Ying Li, Yuanyuan Gao, Yani Zhang, Haisheng Lin, Wen-Xiong Chen

**Affiliations:** Department of Neurology, Guangzhou Women and Children’s Medical Center, Guangzhou Medical University, Guangzhou, Guangdong, China

**Keywords:** MOG antibody associated disease (MOGAD), clinical features, relapse, prognosis, children

## Abstract

**Objective:**

To study the clinical features of children diagnosed with myelin oligodendrocyte glycoprotein antibody-associated disease (MOGAD) in southern China.

**Methods:**

Clinical data of children diagnosed with MOGAD from April 2014 to September 2021 were analyzed.

**Results:**

A total of 93 children (M/F=45/48; median onset age=6.0 y) with MOGAD were involved. Seizures or limb paralysis was the most common onset or course symptom, respectively. The most common lesion locations in brain MRI, orbital MRI, and spinal cord MRI were basal ganglia and subcortical white matter, the orbital segment of the optic nerve, and the cervical segment, respectively. ADEM (58.10%) was the most common clinical phenotype. The relapse rate was 24.7%. Compared with the patients without relapse, relapsed patients had a longer interval from onset to diagnosis (median: 19 days VS 20 days) and higher MOG antibody titer at onset (median: 1:32 VS 1:100) with longer positively persistent (median: 3 months VS 24 months). All patients received IVMP plus IVIG at the acute phase, and 96.8% of patients achieved remission after one to three courses of treatment. MMF, monthly IVIG, and maintaining a low dose of oral prednisone were used alone or in combination as maintenance immunotherapy for relapsed patients and effectively reduced relapse. It transpired 41.9% of patients had neurological sequelae, with movement disorder being the most common. Compared with patients without sequelae, patients with sequelae had higher MOG antibody titer at onset (median: 1:32 VS 1:100) with longer persistence (median: 3 months VS 6 months) and higher disease relapse rate (14.8% VS 38.5%).

**Conclusions:**

Results showed the following about pediatric MOGAD in southern China: the median onset age was 6.0 years, with no obvious sex distribution difference; seizure or limb paralysis, respectively, are the most common onset or course symptom; the lesions of basal ganglia, subcortical white matter, the orbital segment of the optic nerve, and cervical segment were commonly involved in the CNS MRI; ADEM was the most common clinical phenotype; most had a good response to immunotherapy; although the relapse rate was relatively high, MMF, monthly IVIG and a low dose of oral prednisone might effectively reduce relapse; neurological sequelae were common, and possibly associated with MOG antibody status and disease relapse.

## 1 Introduction

Myelin oligodendrocyte glycoprotein (MOG) is found on the myelin surface, and acts as a cellular adhesive molecule to regulate the stability of the oligodendrocyte microtubule (1). In humans, the MOG only is expressed exclusively in the central nervous system (CNS) (2). Antibodies against MOG have been associated with a wide variety of clinical phenotypes, including acquired demyelinating syndromes such as optic neuritis, myelitis, acute disseminated encephalomyelitis (ADEM), neuromyelitis optical spectrum disorder (NMOSD), encephalitis, and aseptic meningitis. Its clinical features and immunopathological mechanisms are distinct from both classic multiple sclerosis (MS) and aquaporin-4 (AQP4)-IgG-positive NMOSD, and being considered as a disease entity in its own, i.e. MOG antibody-associated disease (MOGAD) (3, 4). MOGAD is commonly seen in pediatric patients and can overlap with other autoimmune diseases in the central and peripheral nervous systems (5, 6). In addition, some clinical features of MOGAD differ between children and adults (7). In the present article, we retrospectively investigated 93 pediatric MOGAD patients and focused on their clinical manifestation, radiological presentation, treatment response, relapse rate, maintenance immunotherapy, outcome, and prognosis.

## 2 Article types

Original Research.

## 3 Manuscript formatting

### 3.1 Subjects and methods

#### 3.1.1 Subjects

Children diagnosed with MOGAD from April 2014 to September 2021 in the Department of Neurology of Guangzhou Women and Children’s Medical Center were included. This study was approved by the Ethics Committee of Guangzhou Women and Children Medical Center (Approval No: [2019]40701). Clinical features including demographic data, prodromal events, clinical manifestations, laboratory investigations, neuroelectrophysiological data [electroencephalogram (EEG), visual evoked potential (VEP), brainstem auditory evoked potential (BAEP)], brain magnetic resonance image (MRI), treatment, outcomes, and prognosis were retrospectively reviewed. Neurological disability was assessed by an expanded disability status scale (EDSS). The EDSS was assessed before and after immunotherapy and at the end of follow-up, respectively.

#### 3.1.2 Methods

##### 3.1.2.1 Inclusion criteria

Patients were involved if they were younger than 18 years old and met the diagnostic criteria of MOGAD proposed by Jarius S et al., defined as 1) Monophasic or relapsing acute optic neuritis (ON), myelitis, brainstem encephalitis, or encephalitis, or any combination of these syndromes; 2) MRI or electrophysiological (VEP in patients with isolated ON) findings compatible with CNS demyelination; 3) Seropositivity for MOG-IgG as detected using a cell-based assay employing full-length human MOG as target antigen (8). Encephalitis was diagnosed according to international criteria for inflammatory or infectious encephalitis (9). ADEM was diagnosed according to the criteria proposed by the International Pediatric MS Study Group (IPMSSG) 2013 (10). Acquired demyelinating syndromes were classified according to IPMSSG criteria (10), but MS and NMOSD followed with the more recent criteria (9, 11). An uncategorized MOGAD clinical phenotype was defined as any MOGAD not falling into the clinical phenotype of ADEM, ON, NMOSD, and encephalitis. Anti-NMDAR encephalitis was diagnosed according to the criteria proposed by Graus et al. (12). Acute phases include initial attack at onset and subsequent initial attack at relapses. Relapse was defined as the development of new neurological symptoms one month after the onset of the initial attack or, in the case of ADEM, three months after the onset of the last attack (6, 13, 14), except for the patients with anti-NMDAR encephalitis which were defined as the new onset or symptomatic deterioration occurring after at least two months of improvement or stabilization (15).

##### 3.1.2.2 Exclusion criteria

Patients were excluded if they presented poisoning, infectious, genetic, metabolic, vascular, or neoplastic central nervous system disease, or they failed to complete follow-up.

##### 3.1.2.3 Antibodies test

MOG IgG and AQP4 IgG in serum were detected by the fixed cell-based assay commercial kit (Shaanxi Maiyuan Biotechnology Co., Ltd, Shanxi, China). Anti-NMDAR IgG in serum and cerebrospinal fluid (CSF) were detected by fixed cell-based assay (EUROIMMUN, Lübeck, Germany). These antibodies test was performed by an independent medical agency during acute attacks or follow-up visits. These methods had been reported in detail in our previous study (7, 16). The cut-off value for being MOG positive in the commercial assay was a titer of 1:10.

#### 3.1.3 Neuroelectrophysiological examination

Neuroelectrophysiological examinations including EEG, VEP, and BAEP were performed by neuro-electrophysiologist. The results of EEG, VEP, and BAEP were judged and interpreted by two neuro-electrophysiologists in our hospital. Abnormalities of EEG included: 1) abnormal frequency, amplitude, waveform, distribution, symmetry, stability, and reactivity of the basic rhythm; 2) The amplitude of each frequency band (α, β, θ, δ waves) and the correlation and distribution between the amplitudes are abnormal; 3) Physiological reactions disappear or abnormal reactions appear. 4) Increased slow activity (θ, δ waves); 5) The appearance of pathological waves. Abnormalities of BAEP included: 1) the incubation period of I wave, III wave, and V wave is prolonged; 2) the interphase period of I~ III, III~ V, and I ~ V wave is prolonged; 3) the waveform is poorly differentiated or disappears; 4) the single or bilateral I wave, III wave, and V wave all disappear. Abnormalities of VEP included: 1) the P100 wave disappearing; 2) the latency of the P100 wave prolonging with or without amplitude decrease.

##### 3.1.3.1 Treatment

At the onset and for an acute attack, all patients received intravenous methylprednisolone (IVMP) with a high dose of 15-30 mg/kg/d for 3 to 5 days tailed to oral prednisone (2 mg/kg, reduced 2.5-5 mg every one to two weeks, tapered off within 3 to 6 months or more) in combination with intravenous immunoglobulin (IVIG) administered at 2 g/kg divided into 2-3 days. Patients responding poorly to the IVMP combination with IVIG treatment or severe attack received plasma exchange (n=3) or rituximab (RTX) (n=1) For relapsed patients, maintenance immunotherapy included maintaining mycophenolate (MMF) (0.25 g, once to three times a day), monthly IVIG (400 mg/kg), and maintained a low dose of oral prednisone (initial dose was 2 mg/kg/d, reduced 2.5-5 mg/d every two weeks until dose reached 5 mg/d, 5 mg/d for one to two months and reduced to 2.5 mg/d for two months, and then 2.5 mg every other day for two months) for about 10 to 12 months. The definition described the recovery from onset based on the EDSS score at six months after onset according to Demuth et al. (17) was: classifying the recovery as “complete” if the 6-month EDSS score reached the score before the attack, “partial” if recovery was incomplete, and “absent” if there was no improvement or clinical deterioration.

##### 3.1.3.2 Follow-up

All patients were followed up either by a neurologist in the neurological clinic or by a neurologist *via* telephone contact.

##### 3.1.3.3 Statistical analysis

Statistical analysis was performed using SPSS IBM 20.0. Quantitative data with normal distribution was described by mean ± SD, otherwise median with the interquartile range (IQR). The qualitative data was described by frequency and percentage. Person Chi-Square, Likelihood Ration, or Fish exact test was used to compare the qualitative data. The quantitative data with normal distribution were compared using the independent t-test, otherwise using Mann-Whitney U or Kruskal-Wallis H test. The p-value <0.05 (two-sided) was considered significant. Figures were graphed using GraphPad Prism 7.01 (GraphPad Software Inc., US).

### 3.2 Results

#### 3.2.1 Demographics

A total of 93 children (male: female 45:48) diagnosed with MOGAD came from different regions of southern China, including the provinces of Hunan, Jiangxi, Guangdong, Guangxi, Hainan,and so on. All were of Han Chinese ethnicity. The median onset age was 6.0 years (IQR 4.0-8.0 years). There was no significant difference on onset age between boys and girls [Girl: 6.0 years (IQR 4.0-8.0 years) VS Boy: 6.0 years (IQR 3.5-8.0 years)]. The median interval from onset to diagnosis was 19.0 days (IQR 12.0-28.5 days). There was no significant difference in the median interval from onset to diagnosis between boys and girls [Boy: 20.0 days (IQR 12.0-35.0 days) VS Girl: 19.0 days (IQR 12.0-25.0 days)].

#### 3.2.2 Prodromal events

A slight majority – 51.6% (48/93) – of patients had prodromal events within one month before onset, among which 45.2% (42/93) were infectious events, with acute respiratory infection being the most common (n=40); other events included acute gastroenteritis (n=1) and urinary tract infection (n=1). Four patients had a history of vaccination about 2-14 days before the onset, including live-attenuated oral poliovirus vaccine (n=1), meningococcal meningitis vaccine (n=1), influenza vaccine (n=1), and Streptococcus pneumonia vaccine (n=1). Two patients suffered a head fall before onset. Their head CT scanning showed no abnormality and needed no further treatment.

#### 3.2.3 Neurological symptoms

The most common initial neurological symptom at onset was seizure (22.6%, 21/93), followed by headache (18.3%, 17/93), limb paralysis (17.2%, 16/93), and visual deficits (15.1%, 14/93). The less common initial neurological symptoms included ataxia (9.7%, 9/93), bowel and bladder dysfunction (2.2%, 2/93), speech disorder (2.2%, 2/93), and sensor dysfunction (1.1%, 1/93). While during the whole course, the most common neurological symptoms were limbs paralysis (47.3%, 44/93), followed by seizure (43.0%, 40/93), headache (40.9%, 38/93), visual deficits (31.2%, 29/93), ataxia (29.0%, 27/93), speech disorder (18.3%, 17/93), bowel and bladder dysfunction (15.1%, 14/93), and cranial nerve palsy (10.8%, 10/93). The less common neurological symptoms included respiratory failure due to brainstem involvement (5.4%, 5/93), sensor dysfunction (4.3%, 4/93), and dysphagia (4.3%, 4/93). Bilateral visual deficits were more common than unilateral (20.4% VS 10.8%). Other common symptoms included fever (50.5%, 47/93) and vomiting (29.0%, 27/93).

#### 3.2.4 Laboratory investigation outcome

##### 3.2.4.1 MOG antibody test result

**Figure 1:**
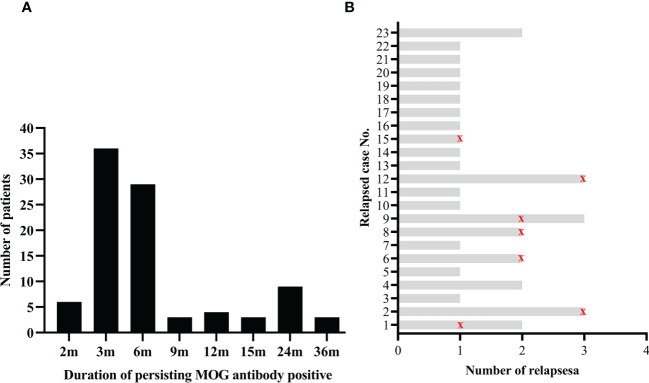
Serum MOG antibody during the disease course. **(A)** Numbers of patients with different duration of persisting MOG antibody positive; **(B)** Status of serum MOG antibody in recurrent patients. The red marks indicate that the serum MOG antibody turned negative during this relapse.

All patients underwent serum MOG antibody tests at onset and acute attack. At the onset, the medium MOG antibody titer was 1:32 (IQR 1:32-1:100). Meanwhile, the serum AQP4 antibody was negative in all patients. At follow-up, patients underwent a MOG antibody test at least on the first, second, and third follow-up per patient every three months after discharge. The median duration of persisting MOG antibody positive was six months (IQR 3-6 months), ranging from 2 to 36 months (**Figure 1A**).

##### 3.2.4.2 Co-existing autoantibodies

Forty-six patients underwent CSF anti-NMDAR antibody tests, and 23.9% (11/46) of them were co-positive for anti-NMDAR antibodies. Seven among these eleven patients have been reported in our previous study (16).

##### 3.2.4.3 CSF test result

All patients underwent CSF examination through lumbar puncture at the onset. The median CSF white blood cell count (WBC) at the first lumbar puncture was 40.0×10^6^/L (IQR 10.0-89.5×10^6^/L), ranging from 1 to 790×10^6^/L, and in which the lymphocyte was dominant. CSF pleocytosis was seen in 72.0% (67/93) of patients. The glucose and chloride levels of CSF were normal. The median protein level in CSF was 0.37 g/L (IQR 0.26-0.50 g/L) and ranged from 0.15 to 2.44 g/L. CSF protein was raised in 47.3% (44/93) of patients. Positive oligoclonal bands (OCBs) was seen in 10.8% (10/93) of patients, including positive in both CSF and serum seen in 8.6% (8/93) of patients and only positive in CSF seen in 2.2% (2/93).

#### 3.2.5 Neuroimaging examination

**Figure 2:**
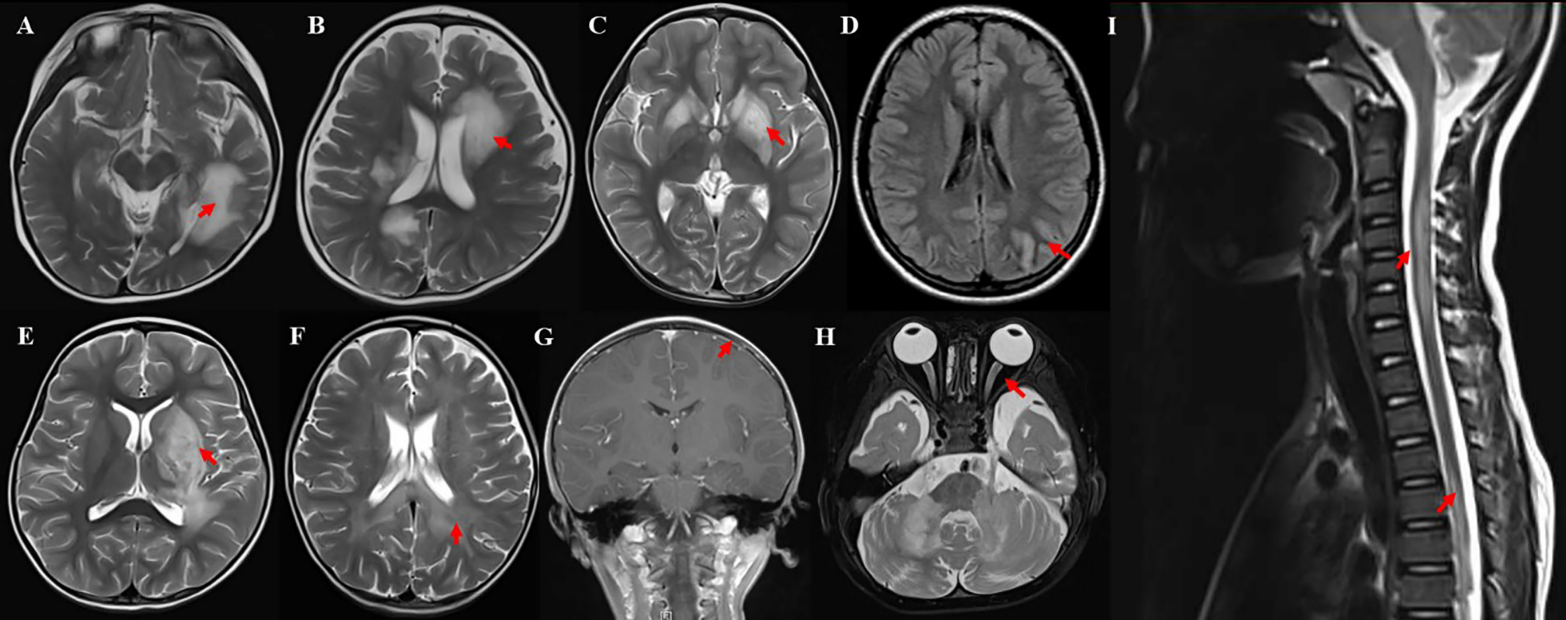
Example of MRI of MOGAD patients. **(A)** Large, poorly demarcated in subcortical white matter; **(B)** MS-like lesion around in periventricular; **(C)** Bilateral basal ganglia obvious edema, similar to EBV encephalitis; **(D)** Left occipital and temporal cortex edema with T2WI-hyperintense lesions along the gyrus; **(E)** Tumefactive lesion in left basal ganglia; **(F)** Symmetric white matter lesions around the posterior horn of lateral ventricle (leukodystrophy-like); **(G)** Local leptomeningeal gadolinium enhancement in T1WI; **(H)** bilateral optic edema; **(I)** longitudinally extensive transverse myelitis in the cervical and thoracic spinal cord.

MRIs were performed for clinical purposes either at acute neurological presentation or at follow-up. All patients underwent brain, orbital, and spinal MRI at onset, and 97.8% (91/93) were abnormal. Meanwhile 39.8% (37/93) of patients showed gadolinium enhancement lesions. In brain MRI, the basal ganglia and subcortical white matter were the common lesion locations, observed in 45.2% (42/93) of patients (**Figures 2A, C**). While cortical gray matter, cerebellum, brainstem, and deep white matter were preferentially involved (**Figure 2D**). However, periventricular white matter, corpus callosum, and leptomeninges were the less frequently involved locations (more details in **Table 1**, **Figures 2B, G**). Nearly three-quarters (72.0%, 67/93) of patients had large (diameter >2 cm) lesions with unclear boundaries in brain MRI, among which three patients had a tumefactive demyelinating lesion (**Figure 2E**), and two patients had a leukodystrophy-like demyelinating lesion (**Figure 2F**). It was also found 19.4% (18/93) of patients had small subcortical patchy lesions with unclear boundaries in brain MRI (**Figure 2A**). In orbital MRI, 19.4% (18/93) of patients were abnormal with optic nerve swelling and gadolinium enhancement (**Figure 2H**). Abnormal Orbital MRI in unilateral was seen in 38.9% (7/18) of patients, and bilateral was seen in 61.1% (11/18). Within the optic nerves, lesions were divided into the five segments with the T2 hyperintensity: orbital (100%,18/18), intra-canalicular (88.9%, 16/18), pre-chiasmal (22.2%, 4/18), chiasmal (11.1%, 2/18), and optic tracts (0.0%, 0/18), with the orbital segment being the most commonly involved. In spinal cord MRI, 21.5% (20/93) of patients were abnormal. Among them, 20.0% (4/20) presented with short myelitis (SM) and 80.0% (16/20) presented with longitudinally extensive transverse myelitis (LETM), in which four patients showed cervical, thoracic, and lumbar spinal cord were involved. The cervical segment was the most frequently involved, followed by the thoracic segment, which mostly involved was the upper thorax (T1-T6) (**Figure 2I**). Four patients had lumbar spinal cord involvement, and two (10%, 2/20) had conus involvement (more details in **Table 1**). Meanwhile, 15.0% (3/20) of these patients had spinal nerve root gadolinium enhancement.

**Table 1:** Clinical features of different clinical phenotypes in pediatric MOGAD.

	ADEM n=54	Encephalitis n=19	ON/NMOSD n=12	Uncategorized MOGAD n=8	*P*
Onset age (median (IQR), years)	5.24(4.0)	8(6.5)	7.5(2.8)	3.8(4.1)	0.007
Clinical presentation					
Fever (N (%))	26(48.1)	15(78.9)	2(16.7)	4(50)	0.005
Headache (N (%))	19(35.2)	12(63.2)	5(41.7)	2(25)	0.142
Seizure (N (%))	18(33.3)	10(52.6)	1(8.3)	1(12.5)	0.029
Visual deficits (N (%))	14(25.9)	2(10.5)	12(100)	1(12.5)	<0.001
Limbs paralysis (N (%))	28(51.9)	7(36.8)	2(16.7)	7(87.5)	0.007
Ataxia (N (%))	18(33.3)	3(15.8)	1(8.3)	5(62.5)	0.026
Laboratory investigation outcome					
MOG antibody titer (median (IQR))	32(68)	32(22)	100(288)	21(200)	0.018
Duration of persistent MOG antibody positive (median (IQR), months)	6(3)	3(3)	6(17)	5(15)	0.031
OCB (Positive, N (%))	5(9.3)	5(26.3)	0(0)	0(0)	0.041
Lesions in MRI					
Cortical gray matter (N (%))	26(48.1)	10(52.6)	1(8.3)	1(12.5)	0.008
Subcortical WM (N (%))	33(61.1)	4(21.1)	4(33.3)	1(12.5)	0.002
Deep WM (N (%))	25(46.3)	0(0)	1(8.3)	1(12.5)	<0.001
Periventricular WM (N (%))	9(16.7)	0(0)	2(16.7)	0(0)	0.043
Corpus callosum (N (%))	8(14.8)	0(0)	0(0)	0(0)	0.026
Basal ganglia (N (%))	33(61.1)	4(21.1)	2(16.7)	3(37.5)	0.002
Brainstem (N (%))	27(50)	0(0)	0(0)	2(25)	<0.001
Cerebellum (N (%))	26(48.1)	0(0)	3(25)	3(37.5)	<0.001
Optic nerve (N (%))	7(13)	0(0)	11(91.7)	0(0)	<0.001
Spinal cord (N (%))	14(25.9)	0(0)	4(33.3)	2(25)	0.013
Leptomeninges (N (%))	2(3.7)	7(36.8)	0(0)	0(0)	0.001
Lesion size (>2cm, N (%))	49(90.7)	9(47.4)	4(33.3)	5(62.5)	<0.001
EDSS at onset (median (IQR))	6.5(2.5)	6(2.5)	5(0.8)	8(2)	0.011
EDSS after acute treatment (median (IQR))	2(1)	1(2)	2(0)	3.5(3.5)	0.009
Follow-up and prognosis					
Duration of follow-up (median (IQR), months)	14(19)	7(10)	12(27)	8(44)	0.134
EDSS at last follow-up (median (IQR))	0(1)	0(1)	1(1)	1(1.5)	0.134
Relapse (N (%))	12(22.2)	5(26.3)	2(16.7)	4(50)	0.084
Sequalae (N (%))	20(37)	6(31.6)	7(58.3)	6(75)	0.097

ADEM, acute disseminated encephalomyelitis; CSF, cerebrospinal fluid; EDSS, Expanded Disability Status Scale; IQR, interquartile range; MOG, myelin oligodendrocyte glycoprotein; MRI, magnetic resonance image; MOGAD, MOG antibody-associated disease; NMOSD, neuromyelitis optica spectrum disorders; OCB, oligoclonal bands; ON, optic neuritis; WBC, white blood cell; WM, white matter.

**Figure 3:**
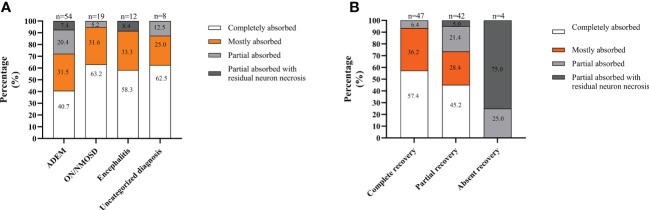
MRI recovery at the last follow-up in the South China cohort. **(A)** Depending on the onset attack phenotype; **(B)** In different recovery groups. The total number of patients in each group is shown above the bar.

Analyzing MRI recovery, at the last follow-up, lesions completely absorbed were observed in 49.5% of patients (46/93), with mostly absorbed in 31.2% (29/93), partially absorbed in 14.0% (13/93), and partially absorbed with residual neuron necrosis in 5.4% (5/93). MRI lesions in the patients with ON/NMOSD or with uncategorized MOGAD were more likely to be completely absorbed. In contrast, lesions in the patients with ADEM were more likely to be partially absorbed with residual neuron necrosis (**Figure 3A**). Moreover, lesions in patients achieving complete recovery were more likely to be completely absorbed. In contrast, lesions in patients absent of recovery were more likely to be partially absorbed with residual neuron necrosis (**Figure 3B**).

#### 3.2.6 Neuroelectrophysiological examination

All patients underwent EEG examination during the acute stage at onset, and 64.5% (60/93) had abnormal EEG. The slow background activity seen in 88.3% (53/60) was the most common abnormality followed by focal epileptiform discharges seen in 25.0% (15/60). Eighty-one patients underwent BAEP examination during the acute stage at onset, and 18.5% (15/81) had abnormal BAEP including prolonged latency of the BAEP waves (n=12) and BAEP waves without differentiation (n=3). Eighty-four patients underwent VEP examination during the acute stage at onset, among which 40 patients younger than 6 years or could not cooperate with the pattern reversal VEP underwent the flash-VEP, and the other 44 patients underwent the pattern reversal VEP. Another 20.0% (8/40) of patients had abnormal flash VEP and 63.6% (28/44) of patients had abnormal pattern reversal VEP. In flash-VEP, bilateral abnormalities were more common than unilateral (n=5 in bilateral VS n=3 in unilateral), so as in pattern reversal VEP (n=18 in bilateral VS n=10 in unilateral). The most common abnormality in flash-VEP was the latency of the P100 wave prolongation with amplitude decrease (50.0%, 4/8), likewise in pattern reversal VEP (39.3%).

#### 3.2.7 Clinical phenotype

At the onset, ADEM (58.10%, 54/93) was the most common clinical phenotype, followed by isolated ON or NMOSD, encephalitis, and the uncategorized MOGAD (more details seen in **Table 1**). Patients with different clinical phenotypes varied in the onset age, clinical and radiological features, MOG antibody titer, duration of persisting MOG antibody positive, the ratio of OCB positive, and EDSS in acute stage at onset and after treatment (more details seen in **Table 1**).

#### 3.2.8 Clinical course and relapse

After a median follow-up of 10 months (IQR 5.0-24.5), 23 (24.7%) of the 93 patients had a relapse and 34 episodes in total. The median follow-up duration for the 23 relapsed patients was 24 months (IQR 8.0-43.0), and the median relapse interval was 12.0 months (IQR 8.0-17.3). For 34 relapsed attacks in total, 79.4% (27/34) of attacks occurred with persistent MOG antibody positive, while only 20.6% (7/34) of attacks occurred when MOG antibody turned negative (**Figure 1B**). Regarding the 34 episodes, ADEM was the most frequently relapsing clinical phenotype observed in 44.1% (15/34), followed by ON 20.6% (7/34), encephalitis 20.6% (7/34), brainstem encephalitis 8.8% (3/34) and other uncategorized MOGAD 5.9% (2/34). 23.5% (8/34) of relapsing episodes occurred post-infection, and 26.5% (9/34) happened during the steroid less than 5 mg/d or within three months after steroid withdrawal. Compared with patients without relapse, relapsed patients had a longer interval from onset to diagnosis, higher MOG antibody titer at onset, longer duration of persisting MOG antibody positive, higher EDSS at last follow-up, and a higher ratio of sequelae (more detail seen in **Table 2**).

**Table 2:** Comparison of clinical features between patients with relapse and without relapse.

	Relapse-free n=70	Relapsed n=23	*P*
Onset age (median (IQR), years)	5.7(4.5)	6(3.5)	0.170
Interval from onset to diagnosis (median (IQR), days)	19(14)	20(350)	0.008
Laboratory investigation outcome			
CSF WBC (median (IQR), x10^6^/L)	40(80)	29(69)	0.786
CSF Protein (median (IQR), g/L)	0.36(0.21)	0.44(0.33)	0.705
MOG antibody titer (median (IQR))	32(90)	100(288)	0.000
Duration of persistent MOG antibody positive (median (IQR), months)	3(3)	24(12)	0.000
OCB (N (%))	8(11.4)	2(8.7)	1.000
Anti-NMDAR antibody (Positive, N (%))	7(10)	4(17.4)	0.456
MRI Lesion size (>2cm, N (%))	49(70)	18(78.3)	0.444
Gadolinium enhancement MRI lesions (N (%))	25(35.7)	12(52.2)	0.162
EDSS at onset (median (IQR))	6.5(3)	6(2.5)	0.676
EDSS after acute treatment (median (IQR))	2(1)	2(1)	0.115
Duration of follow-up (median (IQR), months)	10(17)	24(35)	0.002
EDSS at last follow-up (median (IQR))	0(1)	1(2)	0.009
Sequalae (N (%))	24(34.3)	15(65.2)	0.009

CSF, cerebrospinal fluid; EDSS, Expanded Disability Status Scale; IQR, interquartile range; MOG, myelin oligodendrocyte glycoprotein; MRI, magnetic resonance image; NMDAR, N-methyl-D-aspartate receptor; OCB, oligoclonal bands; WBC, white blood cell.

#### 3.2.9 Treatment and outcome

**Figure 4:**
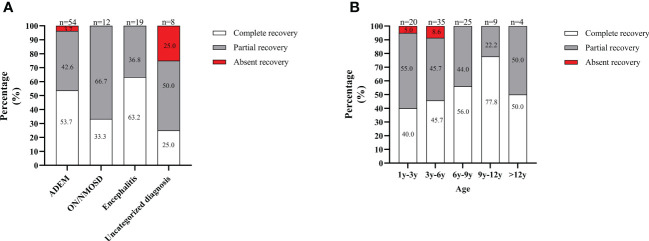
Recovery from the onset attack in the South China cohort. **(A)** Depending on the onset attack phenotype; **(B)** In different age groups. The total number of patients in each group is shown above the bar.

At the onset and for an acute attack at relapse, all patients received IVMP with a high dose of 15-30 mg/kg/d for 3 to 5 days tailed to oral prednisone (2 mg/kg, tapered off within 3 to 6 months or more) combination with IVIG administered at 2 g/kg divided over 2-3 days. The median course of IVMP and IVIG treatment was one (IQR 1-1). Most patients (73.1%, 68/93) achieved remission after only one IVMP and IVIG treatment course. However, 10.8% (10/93) of patients received one IVMP and two IVIG treatment courses and remission. Eight patients received two IVMP and two IVIG treatment courses to achieve remission. Three patients received three IVMP and three IVIG treatment courses to achieve remission. Three patients with severe attacks who responded poorly to IVMP and IVIG treatment received plasma exchanges followed by one course of IVMP and IVIG treatment, subsequent remission. Moreover, one patient did not achieve remission after four IVMP and IVIG treatment courses and responded to rituximab treatment. According to the definition of recovery, 50.5% (47/93) of patients achieved complete recovery at the onset, 45.1% (42/93) achieved partial recovery, and 4.3% (4/93) were absent from recovery. Regarding the onset attack phenotype, patients with ON/NMOSD or ADEM were more likely to achieve complete recovery, while patients with the uncategorized MOGAD were more likely to be absent of recovery (**Figure 4A**). Regarding the onset age, patients aged 9 to 12 years were more likely to achieve complete recovery, while those under six years were more likely to be absent of recovery (**Figure 4B**). For all patients, the median duration of oral prednisone at the first attack was 6.0 months (IQR 4.0-7.0 months) and there was no significant difference in duration of oral prednisone between patients with or without relapse (6.0 months (IQR 4.0-8.0 months) VS 5 months (IQR4.0-6.3 months)), P=0.380).

#### 3.2.10 Maintenance immunotherapy

For 23 relapsed patients, the initial maintenance immunotherapy included maintaining MMF (n=10), monthly IVIG (n=7), and maintaining a low dose of oral prednisone (n=6). Half (5/10) of patients who received MMF treatment became relapse-free with a median follow-up of 32 months (IQR 20.0-39.5 months), while the remaining 50% (5/10) of patients who still relapsed became relapse-free after receiving monthly IVIG treatment with a median follow up for 46 months (IQR 26-25 months). Of patients who received monthly IVIG treatment, 57.1% (4/7) became relapse-free with a median follow-up of 12 months (IQR 7.5-25 months), while the remaining 42.9% (3/7) of patients who still relapsed became relapse-free after receiving MMF treatment with follow-up of 12 months, 18 months, and 30 months respectively. Six relapsed patients received maintenance of a low dose of oral prednisolone for 12.2 ± 1.8 months, three patients became relapse-free with follow-ups of 12 months, 14 months, and 20 months respectively, while the remaining three patients who still relapsed became relapse-free after receiving monthly IVIG treatment with follow-up of 10 months, 15 months, and 18 months. There was no significant difference in the frequency of patients who became relapse-free in these three maintenance immunotherapies used alone (P=1.000).

#### 3.2.11 Prognosis

No patients were lost to follow-up and the median duration of follow-up was 10 months (IQR 5.0-24.5 months). At the last follow-up, 41.9% (39/93) of patients had one to three items of neurological sequelae including movement disorder (25.6%, 10/39), mild visual impairment without affecting daily life (23.1%, 9/39), irritability (12.8%, 5/39), speech disturbance (12.8%, 5/39), learning difficulties (12.8%, 5/39), seizure (10.3%, 4/39), and headache and sleep disorder (7.7%, 3/39). Compared with patients without sequelae, patients with sequelae had higher MOG antibody titer at onset, longer duration of persisting MOG antibody positive, more IVMP treatment courses, higher EDSS after acute treatment and at last follow-up, higher relapse rate, and less ratio of thalamus involvement (more detail seen in **Table 3**).

**Table 3:** Comparison of clinical features between patients with sequelae and without sequelae.

	Non-sequelae n=39	With sequelae n=54	*P*
Onset age (median (IQR), y)	6(3.5)	5(4)	0.111
Interval from onset to diagnosis (median (IQR), days)	19(13)	19(13)	0.330
Clinical presentation			
Seizure (N (%))	15(27.8)	15(38.5)	0.277
Dysphagia (N (%))	2(3.7)	2(5.1)	0.738
Unilateral visual deficits (N (%))	4(7.4)	6(15.4)	0.311
Bilateral visual deficits (N (%))	12(22.2)	7(17.9)	0.614
Limbs paralysis (N (%))	29(53.7)	15(38.5)	0.146
Bowel and bladder dysfunction (N (%))	9(16.7)	5(12.8)	0.609
Brainstem damage symptom (N (%))	3(5.6)	2(5.1)	1.000
Laboratory investigation outcome			
CSF WBC (median (IQR), x10^6^/L)	47(73)	30(89)	0.326
CSF Protein (median (IQR), g/L)	0.37(0.25)	0.34(0.27)	0.564
MOG antibody titer (median (IQR))	32(90)	100(79)	0.009
Duration of persistent MOG antibody positive (median (IQR), months)	3(3)	6(3)	0.010
Anti-NMDAR antibody (Positive, N (%))	8(14.8)	3(7.7)	0.384
Lesions in MRI			
Cortical gray matter (N (%))	21(38.9)	17(43.6)	0.649
Subcortical WM (N (%))	24(44.4)	18(46.2)	0.870
Deep WM (N (%))	15(27.8)	12(30.8)	0.754
Thalamus (N (%))	17(31.5)	5(12.8)	0.037
Basal ganglia (N (%))	23(42.6)	19(48.7)	0.558
Brainstem (N (%))	18(33.3)	11(28.2)	0.598
Optic nerve (N (%))	8(14.8)	10(25.6)	0.192
Spinal cord (N (%))	11(20.4)	9(23.1)	0.754
Lesion size (>2cm, N (%))	38(70.4)	29(74.4)	0.672
Gadolinium enhancement (N (%))	22(40.7)	15(38.5)	0.852
Acute treatment and outcome			
EDSS at onset (median (IQR))	6.5(3.5)	6(3)	0.778
EDSS after acute treatment (median (IQR))	1(2)	2(1)	<0.001
Follow-up			
EDSS at last follow-up (median (IQR))	0(0)	1(1)	<0.001
Relapse (N (%))	8(14.8)	15(38.5)	0.009

ADEM, acute disseminated encephalomyelitis; CRP, C-reactive protein; CSF, cerebrospinal fluid; EDSS, Expanded Disability Status Scale; IQR, interquartile range; IVIG, intravenous immunoglobulin; IVMP, intravenous methylprednisolone; MOG, myelin oligodendrocyte glycoprotein; MRI, magnetic resonance image; NMDAR, N-methyl-D-aspartate receptor; NMOSD, neuromyelitis optica spectrum disorders; OCB, oligoclonal bands; ON, optic neuritis; WBC, white blood cell; WM, white matter.

### 3.3 Discussion

MOGAD is now recognized as a separate disease with distinct clinical and paraclinical features from other CNS inflammation diseases like AQP4 NMOSD and MS (4). Moreover, some clinical features of MOGAD differ between children and adults (7). In the present article, we reported the clinical features and investigation of 93 pediatric MOGAD patients with relatively large case numbers.

Demographically, our patients were similar to the previous studies from other regions in China and other countries in terms of the onset age (6.0 years VS 5.7-7 years) and balanced sex distribution (51.6% female to 49%-57%) (13, 18–22). According to the previous studies, the onset age of pediatric MOGAD patients was younger than patients with AQP4 NMOSD (9.8 years) and MS (14.4 years) (23). While the female ratio of pediatric MOGAD patients was lower than patients with AQP4 NMOSD (71.4%-92.9% female) (24–27) and MS (64% female) (25).

In our study, 51.6% of patients had prodromal events within one month before onset, among which more than 90% were acute respiratory infection and vaccination. Furthermore, the majority of positive pathogenic examination results were viral infections. The ratio of patients with prodromal events was nearly two times higher than that in Zhou et al.’s study, in which it was 26.9% (28). This difference may be caused by the different subjects involved. In our study, we focused on pediatric patients, while pediatric and adult patients were involved in Zhou et al.’s study. Viral illnesses are prevalent among patients and healthy children at the age of MOGAD onset. Whether these infections are occasionally presented in MOGAD or play a role in the pathogenesis of pediatric MOGAD needs further investigation.

We found the top three neurological symptoms initially and during the whole course were seizure, headache, and limb paralysis. Seizure, headache, and limb paralysis are common symptoms of ADEM and encephalitis. Consistent with the distribution of clinical phenotypes, the most frequent phenotype was ADEM (58.1%). The phenotype distribution in our study was similar to other pediatric MOGAD studies (6, 13, 29, 30). However, in contrast to the adult MOGAD, where ON and NMOSD were the common phenotypes, ADEM was less seen (7, 19). In our study, the visual deficit which was the core feature of ON initially and during the whole course was seen in 15.1% and 31.2%, respectively.

The radiological features of MOGAD spontaneously separated from MS but overlapped with AQP4 antibody disease (25). We found basal ganglia was commonly involved and corpus callosum was frequently involved, and the lesions of a majority of patients (72.0%) had unclear boundaries. However, corpus callosum involvement was commonly seen in MS, and basal ganglia and unclear boundaries lesions were uncommonly seen in MS (25). Furthermore, Jurycnzyk et al. found that T1 hypointense lesions, Dawson’s fingers, and ovoid lesions adjacent to the body of lateral ventricles helped discriminate MS from MOGAD (25). The typical lesion locations of MOGAD, including subcortical white matter, cortical gray matter, cerebellum, brainstem, and deep white matter in our study and previous studies, were also common to see in AQP4 disease, except for basal ganglia and thalamus, which was less common in AQP4 disease (13, 25). In orbital MRI, we found bilateral optic nerve involvement was more common than unilateral (61.1% VS 38.9%), and the orbital and intracanalicular segments were frequently involved, similar to previous studies (3, 13, 31–34). In the spinal cord MRI, 21.5% (20/93) of patients were abnormal, with LETM (80.0%) commonly seen and the cervical segment the most frequently involved. LETM was commonly seen in MOGAD, similar to the AQP4 disease but different from MS, in which SM was more common (7, 13, 35–37). Both the thoracic and cervical spinal cord were frequently involved in MOGAD (7, 13, 38, 39). Conus involvement was also more commonly seen in MOGAD (reported in 11%-41%) than in AQP4 disease and MS (39). In our study, 10% of patients had conus involvement. Analyzing MRI recovery at the last follow-up, we found lesions completely absorbed or mostly absorbed in more than 80%, similar to previous studies, showed that resolution of lesions in MRI was more commonly seen in MOGAD, which was not typically seen in AQP4 disease (25, 39, 40). Moreover, we found that the MRI lesion recovery varied in different clinical phenotypes. Lesions in patients with ON/NMOSD or encephalitis were more likely to be completely absorbed. In contrast, lesions in patients with ADEM were more likely to be partially absorbed with residual neuron necrosis regarding the onset attack phenotype.

In our study, the relapse rate was 24.7%, with 34 episodes in total. The relapse rate of MOGAD in previous studies varied from 17.0% to about 95.1% (6, 13, 19, 20, 41–43). Though nearly one-quarter of relapsed patients experience their first relapse within 12 months after onset, the median interval from onset to the first onset varies from 5 to 36 months, and the longest interval even can be longer than ten years (43). Therefore, the follow-up duration must be considered when referring to the relapse rate. In our relapsed patients, 26.5% of episodes occurred during the steroid less than 5 mg/d or within 3 months after steroid withdrawal. Previous studies also found that more than 30% to 40% of patients experienced relapses during steroids on a low dose or withdrawal (33, 40, 41). Moreover, we found that relapsed patients had a longer interval from onset to diagnosis, higher MOG antibody titer at onset, longer duration of persisting MOG positive, and most relapsed attacks occurred with persistent MOG antibody positive. Previous studies also found high MOG antibody titers at onset were associated with increased relapse risk. Furthermore, patients who became MOG antibody negative were less likely to develop relapses than those who remained seropositive (6, 44, 45). Besides, Juryńczyk et al. found persistence of MOG antibodies in children with ADEM phenotype appears to predict further relapses (30). A high titer of species-specific MOG antibodies may be critical for demyelinating effects in mouse and rat animal models (28, 46). These suggest that the MOG antibody might play a pathogenic role in MOGAD. Therefore, it is recommended to tailing to a low dose of oral prednisolone for six months after the initial attack, especially in MOG antibody-positive patients (30).

The treatment of MOGAD is adopted mainly from the AQP4 antibody NMOSD, and much remains unclear (30). IVMP, IVIG, or plasma exchange comprise the first-line immunotherapy for MOGAD. All patients in our study received IVMP and IVIG treatment. Most patients (73.1%, 68/93) achieved remission after only one IVMP and IVIG treatment course. We also found that another 21 patients who received more courses of IVMP or IVIG also achieved remission. Additional IVMP or IVIG treatment courses improve remission, similar to anti-NMDAR encephalitis patients (47). One patient responded poorly to IVIG, and IVMP achieved remission after RTX treatment. In Armangue et al.’s study, three patients also received RTX treatment at disease onset (6). Regarding the recovery from the onset attack, we found that patients with ON/NMOSD or ADEM were more likely to achieve complete recovery. In contrast, patients with uncategorized MOGAD were more likely to be absent from recovery. Furthermore, EDSS at onset and after acute treatment in patients with ON/NMOSD or ADEM tended to be lower than in patients with uncategorized MOGAD. It suggested that the difference in treatment response between the patients with ON/NMOSD or ADEM and uncategorized MOGAD might be caused by the difference in disease severity at the onset.

MMF, monthly IVIG, and maintenance of a low dose of oral prednisone were used as maintenance immunotherapy for relapsed patients in our study. About half of them became relapse-free with only one immunotherapy, and another half became relapse-free after combining with another immunotherapy. Similar to the previous study, MMF, monthly IVIG, and maintenance of a low dose of oral prednisone can decrease the relapse frequency in most patients with MOGAD (6, 34, 48, 49).

Our study found that 41.9% of patients had neurological sequelae with a movement disorder and mild visual impairment. Pediatric MOGAD patients had a better prognosis than adult patients (7). Moreover, visual impairment was common sequelae in pediatric and adult patients but more common in adult patients (7). Furthermore, we found that compared with patients without sequelae, patients with sequelae had higher MOG antibody titer at onset, longer duration of persisting MOG antibody positive, and higher relapse rate. As mentioned above, relapsed patients also had higher MOG antibody titer at onset and longer duration of persisting MOG antibody-positive than relapse-free patients. Jurynczyk et al. found that tailing steroids to a low dose and maintenance helped reduce relapse and improve the prognosis for patients with a severe attack at onset or relapse (30). However, it was unknown whether it could be beneficial for patients with high MOG antibody titer at onset, and the longer duration of persisting MOG antibody.

In summary, our study of 93 pediatric MOGAD patients had a relatively large sample size, with the largest sample size among Chinese pediatric MOGAD studies (7, 13, 20). The demographical features, neurological symptoms, and radiological features were similar to previous pediatric MOGAD studies (3, 6, 13, 18–22, 29–34). However, the ratio of patients who had prodromal events within one month before onset was obviously higher than previous study (28), among which more than 90% were acute respiratory infection and vaccination. Moreover, we found that MRI lesion recovery and recovery from the onset attack varied in different MOGAD clinical phenotypes. In addition, we found that additional IVMP or IVIG treatment courses could improve remission, similar to the anti-NMDAR encephalitis patients (47). The relapse rate was relatively high, and we found that relapsed patients had a longer interval from onset to diagnosis, higher MOG antibody titer at onset, longer duration of persisting MOG positive, and most relapsed attacks occurred with persistent MOG antibody positive. Furthermore, we found that compared with patients without sequelae, patients with sequelae also had higher MOG antibody titer at onset, longer duration of persisting MOG antibody positive, and higher relapse rate. Therefore, it is recommended to tailing to a low dose of oral prednisolone for six months after the initial attack, especially in MOG antibody-positive patients (30). However, it was unknown whether the above-said strategy could benefit those patients with high MOG antibody titer at onset, the longer duration of persisting MOG antibody, or both. Further work is required to identify the optimal therapeutic strategies to reduce relapse and minimize disability in MOGAD patients.

## 4 Conclusions

Of pediatric MOGAD in southern China: the median onset age was 6 years, with no obvious sex distribution difference; seizure or limb paralysis, respectively, being the most common onset or course symptom; the lesions of basal ganglia, subcortical white matter, the orbital segment of the optic nerve, and cervical segment were commonly involved in the central nerve system MRI; ADEM was the most common clinical phenotypes; most had a good response to immunotherapy; the relapse rate was relatively high maybe relation to delay diagnosis and MOG antibody titer status; MMF, monthly IVIG and maintaining a low dose of oral prednisone is effective in reducing relapse; neurological sequelae were not less seen and associated with MOG antibody status, as well as disease relapse.

## Data availability statement

The original contributions presented in the study are included in the article/Supplementary Material. Further inquiries can be directed to the corresponding author.

## Ethics statement

The studies involving human participants were reviewed and approved by the Ethics Committee of Guangzhou Women and Children Medical Center. Written informed consent to participate in this study was provided by the participants’ legal guardian/next of kin.

## Author contributions

XL, WLW, CH and YRZ devised the study concept, handled the acquisition of data, and drafted the manuscript. WXW, LC and YTL aided in the acquisition of data. HZ, YT, BP, KZ, KS and YL analyzed and interpreted the data. YG, YNZ and HL interpreted of data. W-XC helped with study concept, study design, and critical revision of the manuscript for intellectual content. All authors contributed to the article and approved the submitted version.
